# Ultrastructural Morphology of Sperm from Human Globozoospermia

**DOI:** 10.1155/2015/798754

**Published:** 2015-09-08

**Authors:** Giuseppe Ricci, Laura Andolfi, Giuliano Zabucchi, Stefania Luppi, Rita Boscolo, Monica Martinelli, Marina Zweyer, Elisa Trevisan

**Affiliations:** ^1^Institute for Maternal and Child Health, IRCCS “Burlo Garofolo”, 34137 Trieste, Italy; ^2^Department of Medicine, Surgery and Health Sciences, University of Trieste, 34149 Trieste, Italy; ^3^IOM-CNR, SS 14, Km 163, 5 Basovizza, 34149 Trieste, Italy; ^4^Department of Life Sciences, University of Trieste, 34128 Trieste, Italy

## Abstract

Globozoospermia is a rare disorder characterized by the presence of sperm with round head, lacking acrosome. Coiling tail around the nucleus has been reported since early human studies, but no specific significance has conferred it. By contrast, studies on animal models suggest that coiling tail around the nucleus could represent a crucial step of defective spermatogenesis, resulting in round-headed sperm. No observations, so far, support the transfer of this hypothesis to human globozoospermia. The purpose of this work was to compare ultrastructural morphology of human and mouse model globozoospermic sperm. Sperm have been investigated by using scanning and transmission electron microscopy. The images that we obtained show significant similarities to those described in GOPC knockout mice, an animal model of globozoospermia. By using this model as reference, we were able to identify the probable steps of the tail coiling process in human globozoospermia. Although we have no evidence that there is the same pathophysiology in man and knocked-out mouse, the similarities between these ultrastructural observations in human and those in the experimental model are very suggestive. This is the first demonstration of the existence of relevant morphological homologies between the tail coiling in animal model and human globozoospermia.

## 1. Introduction

Globozoospermia represents a rare and severe type of teratozoospermia, first described by Schirren et al. in 1971 [1]. Many animal models have been proposed to investigate pathogenesis of this disorder [2]. Unfortunately, the validity of these models has not been proved by comparison with human pathology. So far, none of the findings observed in these models has been closely compared with human morphological aspects. It has been demonstrated that male mice deleting gene encoding Golgi-associated PDZ- and coiled-coil motif-containing protein (GOPC) are infertile and show globozoospermia with a coiled tail around the nucleus [3]. In this model, coiling tail represents a crucial step of defective sperm maturation resulting in round-headed sperm [4–6]. This process, reasonably, might take place also in human globozoospermia, but, so far, there are no findings supporting this hypothesis. In fact, although coiled tail around the nucleus has been described in globozoospermic subjects since 1974 [7], it has never been recognized as a specifically globozoospermia associated defect. In a recent comprehensive review only 11 out of 50 papers report coiled tails as morphological characteristic of globozoospermia [8]. Moreover, no particular significance has ever been given to it and none of the interpretations of this finding has ever been suggested.

The aim of this study is to compare ultrastructural morphology of human and mouse model globozoospermic sperm.

## 2. Material and Methods

Sperm were obtained from an infertile man with globozoospermia. The initial semen analysis was performed following the standard procedure [9]. Briefly, semen sample was collected by masturbation into sterile containers after 3 days of sexual abstinence and was examined after liquefaction for 30 minutes at 37°C. Semen parameters were assessed using optical microscopy according to World Health Organization guidelines [10]. Leukocyte concentration was determined by standard peroxidase test [11]. The mixed agglutination reaction (MAR) test was used for detecting antisperm antibodies [12]. Ultrastructural studies were also performed. Sperm for scanning electron microscopy (SEM) analysis were washed two times with Dulbecco's modified phosphate buffered saline (PBS) (Sigma-Aldrich, USA) at 500 ×g for 5 min and resuspended in PBS, 3 × 10^6^/mL. One aliquot of the cells suspension was cultured on poly-L-lysine coated coverslips (18 mm diameter) (Menzel Gläser, Braunschweig, Germany) and incubated at 37°C in a 5% CO_2_ atmosphere, for 1 h. Cells were fixed with 2.5% glutaraldehyde in PBS, pH 7.4, at room temperature for 20 min, rinsed in PBS, and postfixed in 1% osmium tetroxide for 30 min. Fixation was followed by rinsing in PBS and then by dehydration through graded ethanol. Samples were transferred to a critical point dryer (Bal-Tec; EM Technology and Application, Fürstentum Liechtenstein) in 100% ethanol and dried through CO_2_. Coverslips were mounted on aluminum sample stubs and gold-coated by sputtering (Edwards S150A apparatus, Edwards High Vacuum, Crawley, West Sussex, UK). SEM micrographs were acquired by a Leica Stereoscan 430i scanning electron microscope (Leica Cambridge Ltd., Cambridge, UK). For preparing samples for optical and transmission electron microscopy, sperm (5 × 10^6^ cells) in PBS were centrifuged at 500 ×g for 5 min and subsequently the pellet was fixed for 30 min at room temperature in a solution of 2.5% glutaraldehyde (Serva, Heidelberg, Germany) in 0.1 M sodium cacodylate buffer (pH 7.4) containing 0.03 M calcium chloride. Samples were washed twice with sodium cacodylate buffer (pH 7.4) and then postfixed with 1% osmium tetroxide for 1 h at 4°C. Postfixed cells were dehydrated with a graded ethanol series ending with 100% ethanol and then embedded in Dow epoxy resin (DER332; Unione Chimica Europea, Milan, Italy) and DER732 (Serva, Heidelberg, Germany). Thin sections for optical microscopy and ultrathin sections for TEM were cut with an Ultrathome III (Pharmacia-LKB, Uppsala, Sweden). Thin sections were stained with toluidine blue at 70°C and observed with a common optical microscope. Ultrathin sections were treated with double staining with lead citrate and uranyl acetate and visualized with a transmission electron microscope (EM208; Philips, Eindhoven, Netherlands).

## 3. Results

**Figure 1:**
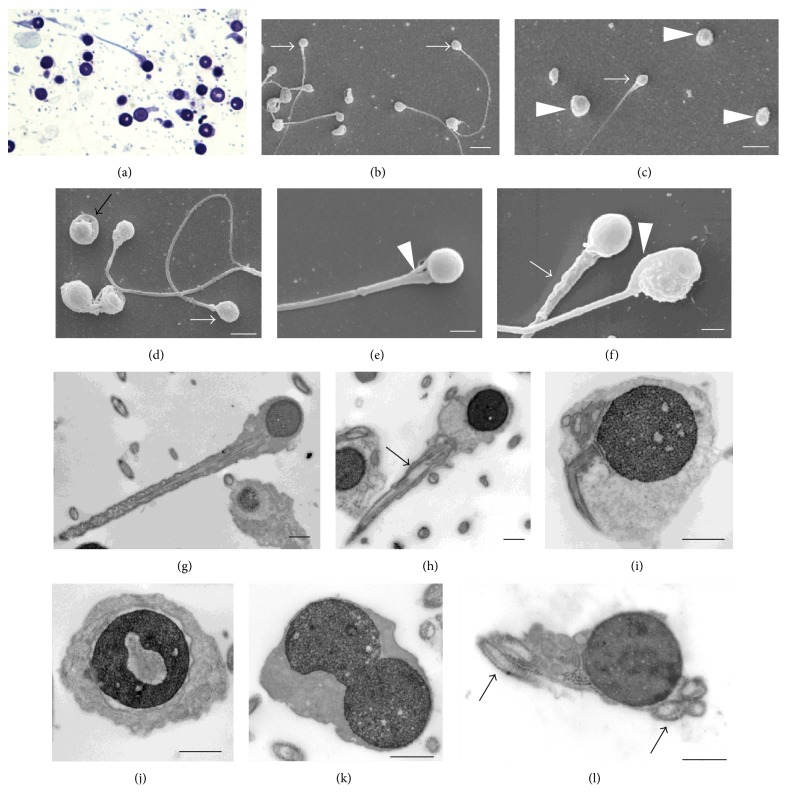
Optical and ultrastructural images of round-headed sperm. (a) Thin section, optical microscopy; ((b), (c), and (d)) SEM view showing round-headed sperm (arrows) and some apparently without tail (arrowheads); (e) a conical neck (arrowhead); (f) a thick midpiece (arrow) and a cord structure at the head side (arrowhead); ((g), (h)) round heads and nuclei, anomalous axoneme in the midpiece (arrow); (i) a bending tail; (j) a detached nuclear envelope and a lacunar chromatin defect; (k) a binucleated head; (l) five axoneme sections enclosed in the same head. ((b) and (c)) White scale bars = 5 *μ*m; (d) white scale bar = 3 *μ*m; ((e) and (f)) white scale bars = 1 *μ*m; ((g), (h), (j), (k), and (l)) black scale bars = 1 *μ*m.

**Figure 2:**
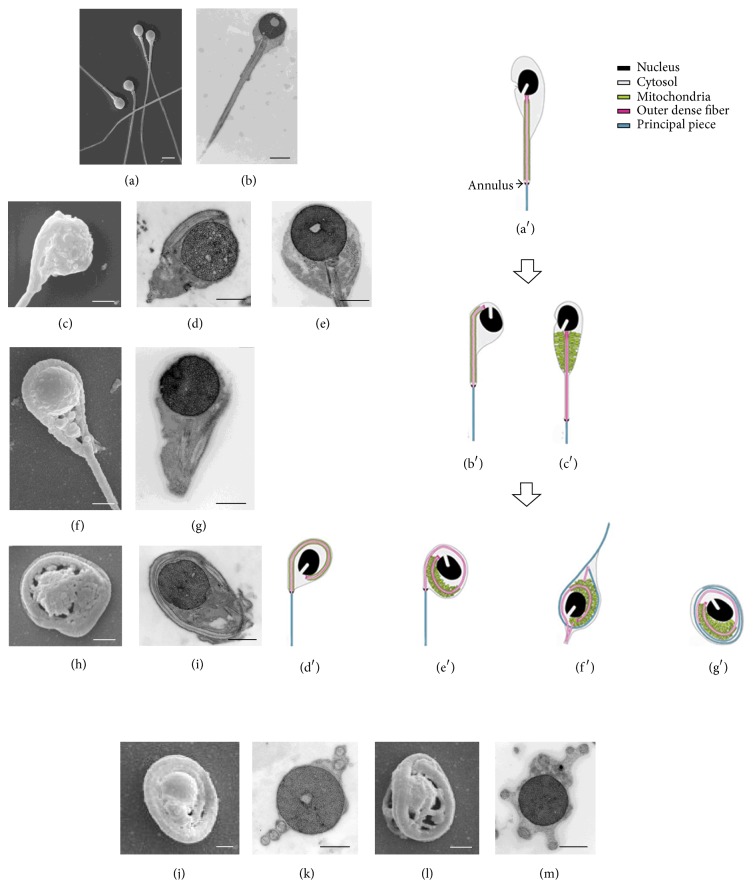
Comparison of sperm tail coiling in human and animal model. ((a) and (b)) SEM and TEM images of straight tail round-headed human sperm; ((c) and (d)) SEM and TEM samples at starting of tail bending; (e) TEM mitochondria accumulated in the conical neck; ((f) and (g)) SEM and TEM tail once turned around the nucleus; ((h) and (i)) tail turned around the nucleus more than once; ((j), (k), (l), and (m)) SEM and TEM images of coiling many times around the nucleus. (a) White scale bar = 2 *μ*m; (b) black scale bar = 2 *μ*m; ((c), (f), (h), (j), and (l)) white scale bars = 1 *μ*m; ((d), (e), (g), (i), (l), and (m)) black scale bars = 1 *μ*m. ((a′), (b′), (c′), (d′), (e′), (f′), and (g′)) Diagrammatic summary of tail disorganizing process in Gopc^−/−^ spermatozoa (see text for the details), modified from Figure  5 of Suzuki-Toyota et al. 2007 [6]. Reproduced with permission from Society for the Study of Reproduction. Copyright 2007 by the Society for the Study of Reproduction, Inc.

The light microscopy semen analysis showed the following characteristics: volume 3.8 mL, sperm count 156 × 10^6^/mL, rapid progressive motility 1%, slow progressive motility 12%, and normal morphology 0%. All spermatozoa had round heads without acrosomal cap, showing complete globozoospermia. Sperm antibody testing with the mixed agglutination reaction (MAR) method was negative for all antibodies. Peroxidase test was negative for leukocytospermia. In order to investigate sperm ultrastructure, SEM and TEM were used, in addition to optical microscopy on thin sections (Figure 1). Optical images showed all nuclei were round. Apparently, no acrosomes were present. The SEM and TEM detailed analysis confirmed round shape of the head. Several sperm were analyzed and several ultrathin sections were obtained, but no acrosomes or buds of acrosome were found. The count of different sperm forms and their percentage were performed on 200 sperm in SEM specimens and on 200 sperm in TEM specimens. As previously reported [8], several sperm anomalies were detected (Figures 1 and 2).

The form most frequently observed was sperm with tail once totally coiled around the nucleus (35%) as in Figures 2(f) and 2(g), like Figure 2(d′). The other forms were represented by the following percentages: 25% of sperm showing the probable start of the tail coiling with the nucleus rolled down the midpiece (in this step a tail segment lay by the nucleus side as in Figures 2(c) and 2(d), quite similar to Figure 2(b′)); 18% of sperm with several tail encirclements of the nucleus (Figures 2(j), 2(k), 2(l), and 2(m), like in Figures 2(f′) and 2(g′)); 15% of sperm with tail coiled around the nucleus more than once (Figures 2(h) and 2(i) as in Figure 2(e′)); 7% of sperm having normally straight tails, as in Figures 2(a) and 2(b), quite similar to Figure 2(a′). Up to five sections of tail per head, both in cross and in oblique/longitudinal section around the nucleus, were frequently observed (Figure 1(l), arrows).

We compared several images of our specimens with the images obtained in a mouse model by Suzuki-Toyota et al. [5, 6]. These authors reproduced in diagram form the supposed steps of the tail disorganizing process in mouse spermatozoa during passage through the epididymis (Figures 2(a′), 2(b′), 2(c′), 2(d′), 2(e′), 2(f′), and 2(g′), modified from Suzuki-Toyota et al. 2007 [6]). Following this hypothesis, we tried to put in a logical order the most frequent aberrant sperm forms observed in our sample. Thus, using the animal model as reference, we were able to identify the probable steps of the tail coiling process in human globozoospermia. Figure 2 summarizes this sequence:

- Sperm having normally straight tails, without tail sections in the perinuclear cytoplasm (Figures 2(a) and 2(b), quite similar to Figure 2(a′)).
- Sperm showing the probable start of the tail coiling with the nucleus rolled down the midpiece, with the membrane covering the tail progressively expanded and joined to the membrane covering the perinuclear cytoplasm. In this step, a tail segment lay by the nucleus side (Figures 2(c) and 2(d), quite similar to Figure 2(b′)).
- Sperm with conical neck filled with mitochondria (Figure 2(e), quite similar to Figure 2(c′)).
- Sperm with tail totally coiled around the nucleus as Figures 2(f) and 2(g) like Figure 2(d′).
- Sperm with tail coiled around the nucleus more than once (Figures 2(h) and 2(i) as in Figure 2(e′)).
- Sperm with several tail encirclements of the nucleus (Figures 2(j), 2(k), 2(l) and 2(m), like in Figures 2(f′) and 2(g′)).

## 4. Discussion

Few studies have focused on sperm tail coiling in human globozoospermia. Pedersen and Rebbe [7] first reported coiling tail in round-headed human spermatozoa, but they did not provide any images and did not mention coiling around nucleus. The first images of sperm with coiled tail around the nucleus in patients with globozoospermia were provided by Italian authors in the 1970s. Baccetti et al. [13] observed sperm with tail coiled around the nucleus but suggested that these cells were a degenerating stage of spermatids with anomalous tail implantation. Castellani et al. [14] noticed that frequently round-headed sperm showed coiled tail around the nucleus but did not give any comments. Other authors, subsequently, reported these abnormalities, but no particular significance was given and no interpretation of this finding has ever been suggested [8]. Therefore, globozoospermia has always been considered as a severe disorder characterized by the presence of round-headed sperm lacking an acrosome, with multiple defects present in a variable percentage of cases. These defects have been variously reported by more than a hundred papers available in the literature [8]. They, generally, have been interpreted as the results of an abnormal maturation process, leading to random and manifold structural aberrations. In this paper, by contrast, we hypothesize that most of the defects of round-headed sperm represent the different stages of a whole disorganizing process and that it is possible to put in a logical sequence several images, apparently not linked to each other. On the basis of the images obtained by TEM and SEM, we suggest that the abnormal maturation process leading to round-headed sperm might include progressive coiling tail around the nucleus, until its final stage where flagellum is many times wrapped around the nucleus.

Our hypothesis has been tested by reviewing the animal models of globozoospermia. Several animal models have been proposed to investigate pathogenesis of globozoospermia (Table 1).

Male mice deleting gene encoding GOPC show globozoospermia with a coiled tail around the nucleus [3]. A straight tail is maintained until the sperm reach the proximal caput epididymis [5, 6]. During passage through the epididymis, the migration of the cytoplasmic droplet containing the nucleus triggers tail disorganization [5, 6]. In this model, tail disorganization is supposed to be caused by weak adhesion between different structures: (1) the plasma membrane and nuclear envelope at the posterior ring, which leads to coiling of the tail around the nucleus; (2) the connecting piece of the flagellum and the implantation fossa of the nucleus, which causes dislocation of the tail from the nucleus and facilitates tail coiling; (3) the mitochondria and outer dense fibers, which results in detachment of the mitochondria from the outer dense fibers. A progressive increase of the tail coiling was observed from proximal to distal caput epididymis [5, 6].

Hrb-deficient mice are infertile and their sperm lack acrosome and present globozoospermia [15]. During spermiogenesis they show nuclear deformity due to invagination and ectopic location of the manchette. The manchette invagination in the nucleus displaces the implantation fossa and causes an alteration of the nucleus-centrioles-axoneme axis, thus causing a loss of sperm cell polarity and altering the direction of flagellar formation, resulting in intracellular flagellum coiling in many mature spermatids [16].

Zpbp1-null sperm exhibit typical morphological defects of globozoospermia such as round-headed appearance and the absence of an acrosome [17]. Zpbp1-null sperm in the proximal epididymis, before the maturation process, carry excessive cytoplasm but not coiled tails. Because the coiling of sperm tails appears during epididymal passage, the authors suggest that reduction in the volume of excessive cytoplasm by the volume regulatory response in the hyperosmotic epididymal fluid causes, likely, retraction and coiling of sperm tails into the excessive cytoplasm surrounding the sperm nucleus. This observation supports the detrimental effect of excessive cytoplasm on sperm maturation.

Sperm acrosome associated 1 (Spaca-1) has recently been found to be a new member of the globozoospermia-related genes [18]. Sperm from Spaca-1-disrupted mice lack acrosome and present globozoospermia. During spermatogenesis, the nuclear plate progressively disappears causing the failure of acrosomal expansion. This leads to the degeneration and disappearance of the acrosome in mature spermatozoa. The authors observe abnormal coiling of the tail around the sperm head during sperm passage through the epididymis. Thus, spermatozoa from the cauda epididymis show a tail coiled around the head [18]. No explanation for these abnormalities has been suggested.

DPY19l2 is a transmembrane protein expressed specifically in spermatids and localized only in the inner nuclear membrane [19]. It has been demonstrated that the absence of Dpy19l2 leads to the destabilization of both the nuclear dense lamina and the junction between the acroplaxome and the nuclear envelope, preventing the anchoring of the acrosome to the nucleus [19]. Recently, deletion of DPY19l2 has been identified in 60–84% of globozoospermic patients of different ethnic backgrounds [20–22]. Dpy19l2 knockout mice show all the characteristics of the human type 1 globozoospermia. In this model, about 20% of sperm presented coiled tails [19]. However, ultrastructural investigation of coiled tails has not been reported. Thus, it is not clear whether the sperm tails are coiled around the nucleus and within the same cellular membrane or they are free below the sperm head.

In other animal models of globozoospermia tail coiling is not present. However, it should be noticed that in these models the sperm are lacking the fundamental criteria of globozoospermia, such as round head and absence of acrosome.

In male mice homozygous for the blind-sterile (bs) mutation, acrosomes do not develop but the nucleus is elongated [23].

Gba2 knockout mice [24] and Pick1 knockout mice [25] produce sperm in which an acrosome, although deformed, can be detected. Interestingly, none of these authors report wrapping of the flagellum around the nucleus.

Csnk2a2^−/−^ mice have also been proposed as a valuable model for studying human globozoospermia [26]. Protein kinase casein kinase II (Ck2) is a cyclic-AMP and calcium- independent serine-threonine kinase. The sperm from mice lacking Ck2*α* show flagellum wrapping around nucleus. However, in a subsequent report, the authors observed that the shape of sperm head was not round but more or less oval, or hooked [27]. Lacking the criteria, the authors concluded that Ck2 deficiency seems to lead to a phenotype different from globozoospermia [27].

On the basis of this review, it can be concluded that only those animal models that show both typical characteristics of globozoospermia, round nucleus and absence of acrosome, present coiled tail around the nucleus (Table 1). Therefore, it can be suggested that tail coiling represents a fundamental characteristic of globozoospermia in animal model. The images that we obtained by TEM and SEM show significant similarities to those described in one of the more convincing animal models above mentioned [3]. Particularly, our images can be sorted in a sequence that seems to reproduce sperm epididymal maturation observed by Suzuki-Toyota et al. (see Figures  4 and 5 of [6]). Although we have no evidence that there is the same pathophysiology in man and knocked-out mouse, the similarities between these ultrastructural observations in human and those in the experimental model are very suggestive and support the hypothesis that abnormal maturation process leading to human round-headed sperm might include progressive coiling tail around the nucleus.

Genetic analysis of our patient was not performed. Therefore, our hypothesis is based only on morphological evaluation. However, it is also supported by observation in patients with a* DPY19L2* deletion [19]. Pierre et al. have shown that the microscopic aspect of sperm from* DPY19L2* homozygously deleted men and those of spermatids from knockout mice was remarkably similar [19]. In fact, by using light microscopy analysis they observed both in human and animal model the same morphological defects, including complete or partial absence of the mitochondrial sheath, coiled tails, several flagella, and bicephalic sperm [19].

We found that a minority of sperm (7%) showed a normally straight tail. It can be hypothesized that, in these sperm, tail coiling process has not yet started, similar to what was described in the animal model [6]. It should be noticed that the mouse model evaluates epididymal sperm in different maturation stages until mature spermatozoa [6], whereas our study has been conducted on ejaculated spermatozoa. However, other authors have reported that sperm from subjects with globozoospermia show relevant morphological similarities to epidydimal sperm from knockout mice [19].

The precise mechanism of the coiling of the flagellum around the nucleus is not known yet. Sperm with coiled tail around the head were found in 91% of infertile patients, although at very low percentages (3%) [28]. The significance of such abnormal sperm is unclear since their percentage in the ejaculate was independent of age, smoking, sperm quantity, hormonal status, seminal biochemical markers, varicocele, and male tract infection [28]. Testicular spermatozoa with tail coiled around the head were observed within the seminiferous tubules by using Nomarski optics in both live and fixed preparations from control men [29]. Our observations do not allow clarifying either the origin or the driving force of tail coiling in human globozoospermia. However, it can be hypothesized that, similar to mouse model [6], tail coiling is related to the migration of the cytoplasmic droplet from the neck to the distal end of the midpiece and that dislocation of the connecting piece from the nucleus accelerates tail coiling. As it has been shown in animal model [6], the weakness in the adhesion between the connecting piece and the implantation fossa of the nucleus might have a fundamental role in the pathogenesis of tail coiling also in human. Further studies are needed to elucidate this very complex biological process.

In conclusion, to the best of our knowledge, this is the first demonstration of the existence of relevant morphological homologies between the tail coiling in animal model and human globozoospermia. Therefore, the significance of sperm tail coiling in human globozoospermia should be reconsidered. Our observations further support the validity of animal model for investigating the pathogenesis of this rare particular disorder and should encourage further research in this direction.

## Figures and Tables

**Table 1:** Knockout mouse models of globozoospermia.

Mouse model	Protein name	Round head	Acrosome	Coiled tail around the nucleus	Reference
*Gopc*	Golgi-associated PDZ and coiled-coil motif-containing protein	Yes	Absent	Yes	[3]

*Agfg1 (Hrb) *	ArfGAP with FG repeats 1	Yes	Absent	Yes	[15]

*Zpbp *	Zona pellucida binding protein	Yes	Absent	Yes	[17]

*Spaca-1*	Sperm acrosome associated 1	Yes	Absent	Yes	[18]

Dpy19l2	Dpy-19-like 2 (*C. elegans*)	Yes	Absent	?	[19]

*AKR-bs X 129*	?	No (elongated)	Absent	No	[23]

*Gba2*	Beta glucosidase 2	Yes	Present (deformed)	No	[24]

*Pick-1*	Protein interacting with C kinase 1	Yes	Present (deformed)	No	[25]

*Csnk2a2*	Casein kinase 2, alpha prime polypeptide	No (oval)	Present (deformed)	Yes	[26]
